# Sex Differences are Reflected in Microstructural White Matter Alterations of Musical Sophistication: A Diffusion MRI Study

**DOI:** 10.3389/fnins.2021.622053

**Published:** 2021-07-22

**Authors:** Mohammad-Mehdi Mehrabinejad, Parnian Rafei, Hossein Sanjari Moghaddam, Zeinab Sinaeifar, Mohammad Hadi Aarabi

**Affiliations:** ^1^Faculty of Medicine, Tehran University of Medical Sciences, Tehran, Iran; ^2^Department of Psychology, Faculty of Psychology and Education, University of Tehran, Tehran, Iran; ^3^Department of Neuroscience, Padova Neuroscience Center (PNC), University of Padova, Padova, Italy

**Keywords:** musicality, musical sophistication, diffusion magnetic resonance imaging, connectometry, white matter, white matter microstructural changes, music perception and cognition

## Abstract

**Background:** The human-specified ability to engage with different kinds of music in sophisticated ways is named “Musical Sophistication.” Herein, we investigated specific white matter (WM) tracts that are associated with musical sophistication and musicality in both genders, separately, using Diffusion MRI connectometry approach. We specifically aimed to explore potential sex differences regarding WM alterations correlated with musical sophistication.

**Methods:** 123 healthy participants [70 (56.9%) were male, mean age = 36.80 ± 18.86 year], who were evaluated for musical sophistication using Goldsmiths Musical Sophistication Index (Gold-MSI) self-assessment instrument from the LEMON database, were recruited in this study. The WM correlates of two Gold-MSI subscales (active engagement and music training) were analyzed. Images were prepared and analyzed with diffusion connectometry to construct the local connectome. Multiple regression models were then fitted to address the correlation of local connectomes with Gold-MSI components with the covariates of age and handedness.

**Results:** a significant positive correlation between WM integrity in the corpus callosum (CC), right corticospinal tract (CST), cingulum, middle cerebellar peduncle (MCP), bilateral parieto-pontine tract, bilateral cerebellum, and left arcuate fasciculus (AF) and both active engagement [false discovery rate (FDR) = 0.008] and music training (FDR = 0.057) was detected in males. However, WM integrity in the body of CC, MCP, and cerebellum in females showed an inverse association with active engagement (FDR = 0.046) and music training (FDR = 0.032).

**Conclusion:** WM microstructures with functional connection with motor and somatosensory areas (CST, cortico-pontine tracts, CC, cerebellum, cingulum, and MCP) and language processing area (AF) have significant correlation with music engagement and training. Our findings show that these associations are different between males and females, which could potentially account for distinctive mechanisms related to musical perception and musical abilities across genders.

## Introduction

The human-specified ability and skills to engage with different kinds of music in ways with different sophistication degrees is named “Musical Sophistication” (Merriam and Merriam, 1964; Müllensiefen et al., 2014). There is a long scientific history for the assessment of music abilities and behavior and music perception in humankind (Boyle and Radocy, 1987; Rickard et al., 2015), and measuring musicality and musical sophistication, and their correlates had always been a challenge in different research paradigms.

The history of assessing musical sophistication goes back to a hundred years ago since the first measurement of musical talent was developed by Carl Seashore and colleagues in 1919 (Seashore et al., 1956). However, the majority of assessments employed for assessing musical ability (i.e., musicality) of individuals have been carried out in specific populations such as professionals in the field of music (e.g., musicians) or those who suffer from a pathological musical condition (e.g., amusia) (Brockmeier et al., 2011; Law and Zentner, 2012; Sato et al., 2015; Larrouy-Maestri et al., 2019). This vast omission in musicality testing paradigms led to the development of the recently developed batteries such as the Goldsmiths Musical Sophistication Index (Gold-MSI) self-assessment instrument (Müllensiefen et al., 2014).

The Gold-MSI is a self-report measure consisting of five subscales and two subjective auditory listening tests (a melodic memory task and beat perception task), as well as an exercise regarding a sound similarity judgmental task (Müllensiefen et al., 2014; Baker et al., 2020). Musical sophistication in the Gold-MSI paradigm is conceptualized as a psychometric construct that is involved in developing musical skills, achievements, and expertise and defines a multi-faceted musical behavior in individuals (Müllensiefen et al., 2014). As a result of the aforementioned attributes, the Gold-MSI questionnaire has been shown to be suitable for assessing musicality in the non-musician population in a comprehensive way (Baker et al., 2020). Moreover, this instrument is capable of assessing a broad range of musical-related abilities, consisting of the individual’s performance on a musical instrument, their listening expertise, the ability to engage with music in functional settings or to communicate about music (Müllensiefen et al., 2014).

There are a number of neuroimaging studies that have investigated the neural basis, functional neural connectivity, and neuro-anatomical evidences of musical perception and expertise in grey matter (GM) of the humankind brain (Parsons, 2001; Schmithorst and Wilke, 2002; Elmer et al., 2013; Wu et al., 2013; Oechslin et al., 2018; van Vugt et al., 2021). In general, they have identified significant pieces of evidence on differences in the brain GM in brain areas such as the cerebellum (Gaser and Schlaug, 2003), Broca’s area (Sluming et al., 2002), and Planum temporal (Keenan et al., 2001; Burkhard et al., 2020) in musicians and non-musicians. Several studies also demonstrate that brain structures with a close connection with the motor system have significant association with musicality (Schlaffke et al., 2020; Møller et al., 2021).

However, the mainstream studies in this field are majorly focused on the structural and functional characteristics of the GM in professional musicians, and the WM correlates of musicality is hitherto considered as a relatively understudied phenomenon in this field. In general, empirical pieces of evidence show that musicians exhibit changes in the white matter structure of their brain (Levitin, 2012). For instance, the Corticospinal Tract (CST) has been shown to have reduced fractional anisotropy (a measure of the directionality of water diffusion) in professional musicians, which indicates increased radial diffusivity (Imfeld et al., 2009). This notion is, however, discrepant among published studies investigating the diffusivity measures between higher and lower fractional anisotropy values of known tracts in response to musical training (Schlaug, 2015). These variations and inconsistencies in such findings have been reported to be potentially influenced by factors such as fiber density, cell membrane density, axon collateral sprouting, axon diameter, myelination, and fiber coherence. Higher fractional anisotropy values has been thought to reflect more aligned fibers in a specific tract, whereas lower fractional anisotropy values indicate less alignment of fibers in addition to more axonal sprouting and more branching of axons close to the cortical target region (Wan et al., 2014; Rüber et al., 2015; Schlaug, 2015). Moreover, the macro and microstructural organization of the Arcuate Fasciculus (AF)—a prominent WM tract connecting temporal and frontal brain regions— has been shown to have a predictive role in learning rate and learning speed in musical tasks related to rhythm and melody training (Vaquero et al., 2018). Other studies suggest that musical training is associated with microstructural adaptations in the AF, which appear as increased tract volume in the right AF of musicians compared to non-musicians (Halwani et al., 2011). A more recent study found that the microstructural organization of WM tracts that connect auditory and frontal motor regions in both hemispheres of the brain may serve as a neural foundation of the musicality or musicians’ advantage (Li et al., 2021). Nevertheless, the most frequently reported WM microstructural differences in musicians compared with non-musicians appears to be in the cross-hemispheric connections (i.e., Corpus Callosum CC) (for a review, see Moore et al., 2017).

In light of the small number of studies investigating the WM alterations related to musicality and given the conflicting results that former studies presented, we aimed to investigate the brain white matter (WM) integrity to identify the microstructural patterns associated with musicality in general population.

Diffusion MRI Connectometry approach, the analysis method that we used in this study, is a novel approach, is reported to be more sensitive than diffusion tensor imaging (DTI) metrics (Yeh et al., 2016), and gives additional spatial resolution to track the WM fibers (Rahmani et al., 2017; Moghaddam et al., 2020; Mehrabinejad et al., 2021). Therefore, in the present study, we investigated specific WM tracts associated with musical sophistication and musicality utilizing diffusion MRI connectometry as an exploratory approach and looked for any variations in WM microstructural patterns related to demographic characteristics such as gender, a predictor of self-reported musical sophistication (Gold-MSI) subscales (Greenberg et al., 2015).

We hypothesized that those WM tracts that were formerly found to be associated with auditory and motor regions, such as AF, may be significantly correlated with two Gold-MSI subscales (active engagement and music training). To the best of our knowledge, no previous study has specifically examined the WM correlates of musical sophistication using the diffusion MRI connectometry approach. In addition, we were interested in exploring potential sex differences related to the association between WM trajectories and each Gold-MSI subscale. Given the empirical evidence showing significant within-hemisphere and inter-hemisphere differences between males and females’ brain structural and functional connectomes using DTI (Ingalhalikar et al., 2014), and the gap in the literature in terms of sex differences in musical sophistication and its neural correlates, we sought potential differences by running discrete analyses for male and female subjects of this study.

## Materials and Methods

### Overview

To recognize the similarity in local connectivity patterns and identify the pathways of WM tracts, diffusion MRI connectometry approach measures the density of water diffusion through different directions of a voxel. Thus, water diffusivity measurement which is the speed of water diffusion in different directions, and is the primary concern of conventional DTI analysis, is replaced by measuring the density of water diffusion in diffusion MRI connectometry; which leads us to the identification of the local connectivity of fibers and tracking the subcomponents of the tract pathways which are significantly associated with our study variables (i.e., musical sophistication).

### Study Data

In the current study, we obtained all the required data from the “Leipzig Study for Mind-Body-Emotion Interactions” (LEMON) dataset (Babayan et al., 2019)^1^. The LEMON study was carried out in four series from September 2013 to September 2015. After prescreening via telephone interview, participants who met the eligibility criteria were invited to Max Planck Institute for Human Cognitive and Brain Sciences (MPI-CBS) for further evaluations. Exclusion criteria were present or past history of any cardiovascular (hypertension, congenital heart disease, or heart attacks), psychiatric [conditions needing more than 2 weeks therapy within last 10 years, post-traumatic stress disorder (PTSD), psychosis, or suicidal attempts], neurological [e.g., multiple sclerosis (MS), epilepsy, stroke], or malignant diseases, and also some particular medication usage (e.g., centrally active drugs, cortisol, alpha- or beta-blocker, extensive alcohol, benzodiazepine, cocaine, amphetamines, cannabis, or opiates) as well as any MRI contraindications.

A total of 227 eligible German-speaking participants who were screened via a telephone interview in day 0, participated in a 5-day survey. All participants were examined at the Day Clinic for Cognitive Neurology of the University Clinic Leipzig and the MPI-CBS in Leipzig, Germany. Briefly, all enrolled participants were asked to complete: (1) four fMRI and one structural scan in one session; (2) a battery of personality questionnaire; and (3) a set of cognitive, attention, and creativity related tasks. The Gold-MSI questionnaire was given in the first day of the study.

### Participants

Of 227 participated individuals in the LEMON study, 123 healthy participants, who were evaluated for musical sophistication using Gold-MSI from the LEMON database, were recruited in this study. The present study was carried out in accordance with the World Medical Association Declaration of Helsinki revised in 1989 and approved by the Ethics Committee of the University of Leipzig (reference number 154/13-ff).

### Goldsmiths Musical Sophistication Index (Gold-MSI)

The Gold-MSI evaluates self-reported musical abilities and behaviors on multiple aspects in the general population (Müllensiefen et al., 2014). Five subscales of musical sophistication consist of active musical engagement, self-reported perceptual abilities, musical training, self-reported singing abilities, and sophisticated emotional engagement with music (Müllensiefen et al., 2014). Of five main subscales (active engagement, musical training, perceptual abilities, singing abilities, and emotions) only first two subscales were included in the LEMON study.

Active engagement is defined as the level of music engagement including reading, writing, and listening to music as well as the time and income spent on music and music events attendance (Müllensiefen et al., 2014). Musical training reveals the musical dedication according to the time (peak hour per day and amount of training years) spent on training and number of instruments played (Müllensiefen et al., 2014). A subset of 16 items, scoring on a seven-point Likert scale (1: “absolutely disagree” to 7: “absolutely agree”) were measured. Higher scores are attributed to higher musical sophistication. The German version of this scale was used in this study (Schaal et al., 2014).

### Image Acquisition

Magnetic resonance images were acquired with a 3 Tesla scanner (MAGNETOM Verio, Siemens Healthcare GmbH, Erlangen, Germany) and a 32-channel head coil in addition to a multi-band accelerated sequence combined with an in-plane GRAPPA (TR = 7,000 ms, TE = 80 ms, GRAPPA acceleration factor = 2, bandwidth = 1,502 Hz/Px, field of view = 220 × 220 mm^2^, and voxel size = 1.7 × 1.7 × 1.7 mm^3^) aiming for diffusion MRI data collection. Consequently, seven b0 and sixty diffusion MRI images were recorded.

### Imaging Data and Statistical Analysis

Preprocessing steps, i.e., head motion, eddy current distortions, and susceptibility artifacts because of the magnetic field inhomogeneity correction, were carried out using the ExploreDTI toolbox^2^ (Leemans et al., 2009). Diffusion data were reconstructed within Montreal Neuroimaging Initiative (MNI) space, using q-space diffeomorphic reconstruction (Yeh et al., 2011) to obtain the Spin Diffusion Function (SDF; main component of diffusion connectometry). Subsequently, a diffusion sampling length ratio of 1.25 was used.

Diffusion metrics association with two subscales of Gold-MSI (active engagement and musical training) were analyzed through dMRI connectometry (Yeh et al., 2016). A multiple regression model was performed to consider these subscales in female and male participants, separately. Age and handedness were taken into account in all the analyses to adjust for possible confounding effects. T-score threshold of 2.5 was defined to delineate local connectomes. Deterministic fiber tracking algorithm was used to estimate WM tracts (Yeh et al., 2013). After normalization, topology-informed pruning was undertaken to prevent false positive tracking. Tracks were generated from bootstrap resampling and a length threshold of 20 voxel distance was utilized to highlight tracks. The seeding number for each permutation testing was set to 100,000. A total of 2,000 randomized permutations were employed to the group label to obtain the null distribution of track lengths in order to estimate the false discovery rate (FDR).

## Results

dMRI of 123 healthy participants underwent analysis to investigate the association between musical sophistication subscales with WM connectivity in different genders. Of those, 70 (56.9%) were male, 120 (97.6%) were right-handed, and were on average middle-aged adults (mean age ± standard deviation (SD): 36.80 ± 18.86 year) (Table 1).

**TABLE 1:** Demographic characteristics of study participants (*n* = 123).

**Age**	36.80 ± 18.86 (years)

**Gender**	Male: 70 (56.9%)
	Female: 53 (43.1%)
**Handedness**	Right-handed: 120 (97.6%)
	Left-handed: 0 (0%)
	Ambidextrous: 3 (2.4%)

The mean score of active engagement and musical training were 23.95 ± 8.36 (range: 7–44) and 19.65 ± 10.09 (range: 7–40), respectively. Males and females did not differ significantly in their active engagement and musical training scores (*p* = 0.45 and 0.80, respectively) (Table 2).

**TABLE 2:** Gold-MSI results for active engagement and musical training subscales in all participants and each gender separately.

**Variables**	**Total (Mean (*SD*))**	**Male (Mean (*SD*))**	**Female (Mean (*SD*))**	***p*-value**
**Active engagement**	23.95 (8.36)	24.44 (8.41)	23.30 (8.33)	0.45
**Musical training**	19.65 (10.09)	19.46 (10.28)	19.91 (9.92)	0.80

dMRI connectometry analysis revealed significant correlations (either positive or negative) between quantitative anisotropy (QA) of some specific WM trajectories with each Gold-MSI subscales in different genders (Table 3).

**TABLE 3:** WM tracts with significant association with Gold-MSI subscales in each gender.

**Variables**	**WM tracts**	
	**Positive**	**Negative**
**Active engagement**		
**Male**	Genu, body, and splenium of CC, bilateral parieto-pontine tract, right CST, MCP, bilateral cortico-thalamic pathway, right fronto-pontine tract, right cingulum, bilateral cerebellum, and left AF	None
**Female**	None	Body and splenium of CC
**Musical training**		
**Male**	Genu, body and splenium of CC, bilateral cingulum, right CST, bilateral parieto-pontine tract, and left AF	None
**Female**	None	Genu, body, and splenium of CC

**FIGURE 1:**
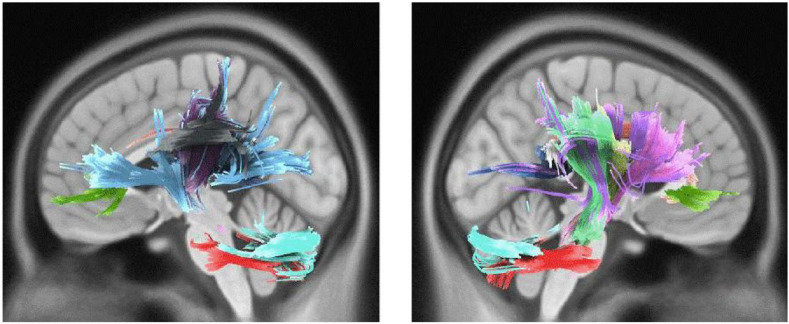
White matter pathways with significantly positive association with Active engagement in male participants (FDR = 0.008).

**FIGURE 2:**
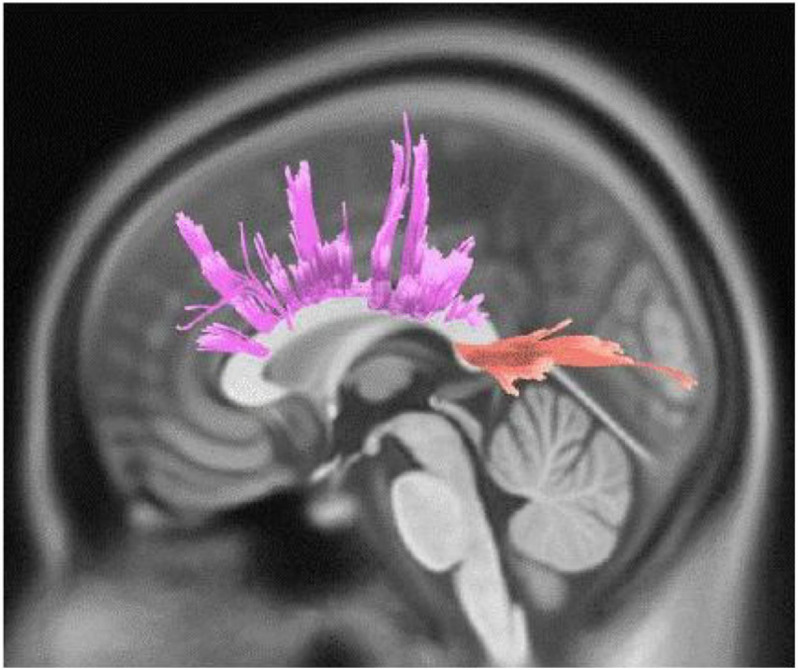
White matter pathways with significantly negative association with Active engagement in female participants (FDR = 0.046).

Active engagement was significantly and positively correlated with WM integrity in genu, body, and splenium of CC, bilateral parieto-pontine tract, right cortico-spinal tract (CST), middle cerebellar peduncle (MCP), bilateral cortico-thalamic pathway, right fronto-pontine tract, right cingulum, bilateral cerebellum, and left arcuate fasciculus (AF) in male participants (FDR = 0.008, Figure 1). Besides, it was significantly, but negatively, correlated with QA values in body and splenium of the CC in females, as well (FDR = 0.046) (Table 3 and Figure 2).

**FIGURE 3:**
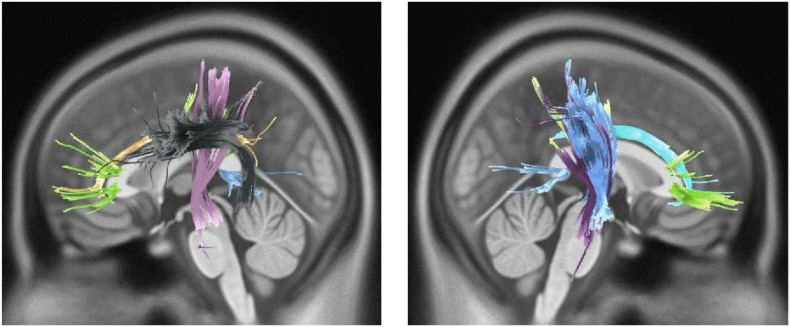
White matter pathways with marginally significantly positive association with musical training in male participants (FDR = 0.057).

**FIGURE 4:**
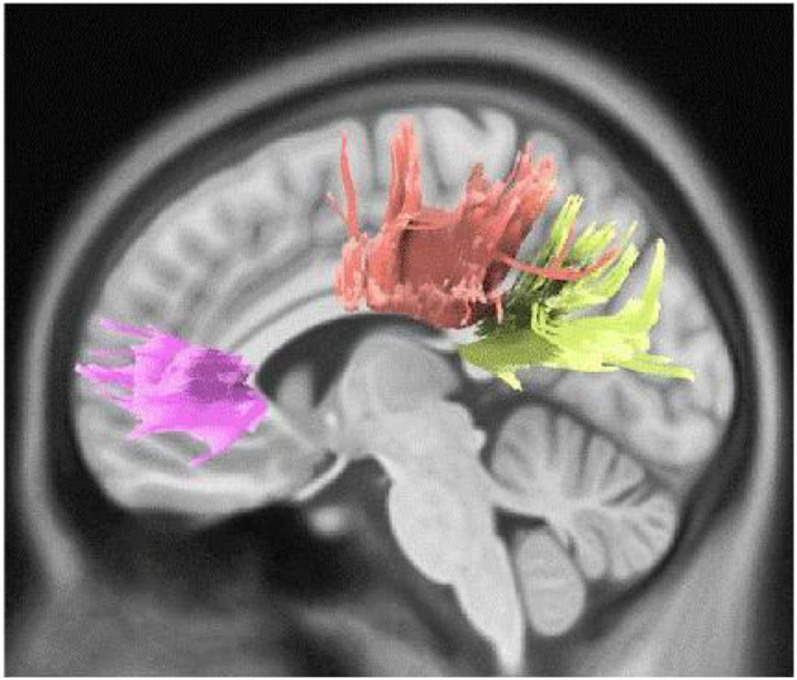
White matter pathways with significantly negative association with musical training in female participants (FDR = 0.032).

We also observed direct yet marginally significant association between musical training and QA values of genu, body and splenium of CC, bilateral cingulum, right CST, bilateral parieto-pontine tract, and left AF in males (FDR = 0.057, Figure 3). Besides, inverse association was detected in genu, body and splenium of CC in females (FDR = 0.032) (Table 3 and Figure 4).

## Discussion

Alterations in WM architecture in individuals with high musical abilities is a common report across studies probing into the association between WM characteristics and musical abilities. However, the connection of musical sophistication, which is a broad concept of musical-related abilities in the population of non-professional musicals, with the WM microstructures has been overlooked. Thus, in the current study, we explored the association of musical sophistication and abilities related to musicality and the WM microstructures in the brain of a population with a diverse musical training and engagement background. In order to do this, we assessed the WM correlates of active engagement and musical training as the two Gold-MSI subscales. In general, we showed that the WM microstructure connectivity pattern correlates with the Gold-MSI subscales of musical sophistication and gender. To elaborate on, our findings support our hypothesis regarding the sex differences in terms of WM microstructural alterations related to musical active engagement and musical training. Within the male participants, results exhibited higher WM coherence in CC, CST, MCP, AF, cingulum, and parietopontine tract with a higher score in both investigated subscales of Gold-MSI (i.e., active engagement and musical training) and higher integrity in the cortico-thalamic tract, fronto-pontine tract, and cerebellum with higher active engagement, whereas findings related to the female participants showed a negative correlation between WM connectivity in CC and both investigated Gold-MSI subscales.

**FIGURE 5:**
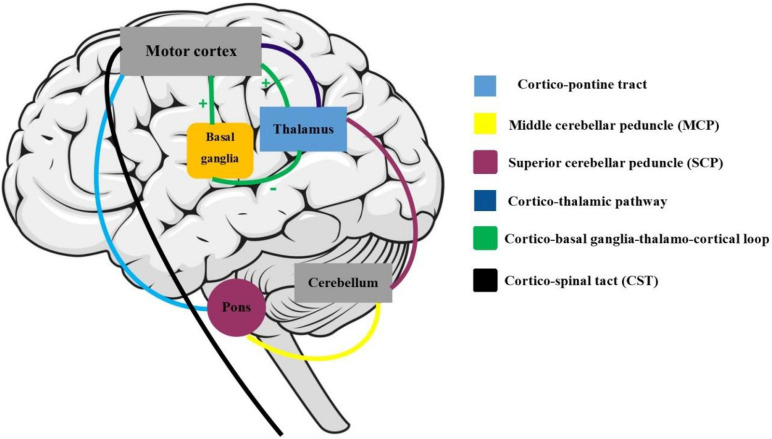
Motor and somatosensory system: the maturation of cortico-spinal tract (CST) is responsible for smooth and fine movements of limbs. The basal ganglia and cerebellum modulate the CST functions through the afferent (cortex, brain stem, and spinal cord) and efferent (through the thalamus) fibers. The cortico-basal ganglia-thalamo-cortical circuit is a modulator motor system with inhibitory and excitatory inputs.

Multiple efforts have been taken to underpin the neural basis of musical perception in both WM and GM (Parsons, 2001; Schmithorst and Wilke, 2002; Elmer et al., 2013; Wu et al., 2013; Oechslin et al., 2018). For instance, Heschl’s gyrus in the primary auditory cortex has indicated to be a possible marker of musicality due to a MEG study comparing professional and amateur musicians with non-musicians which showed a significant difference in MEG activity between the groups in the primary auditory cortex, moreover, a brain volumetric difference has also been found between groups (Schneider et al., 2002). Results have shown that there are significant pieces of evidence on differences in the brain GM in brain areas such as the cerebellum (Gaser and Schlaug, 2003), Broca’s area (Sluming et al., 2002), and Planum temporal (Keenan et al., 2001; Burkhard et al., 2020) in musicians and non-musicians. Nevertheless, the direct findings related to WM are limited, and the interpretation of our results requires discussing our findings based on previously known functions of the structures, as follows:

Active engagement and musical training are defined as the engagement and dedication level with musical activities and musical events (Müllensiefen et al., 2014). Both these two subscales have a close association with motor and somatosensory activities. For instance, active musical engagement has been defined as the amount of time and effort spent on music, whereas the musical training refers to the formal amount of musical training received. Both these functions are involved with motor activities regardless of the specific musical activity or instrument that one may employ. As described further, our findings are in accordance with the previously known functions of the motor cortex, which regulates the execution and control of movements. The motor cortex is mainly divided into five cortical regions in two main brain lobes, including the frontal lobe (primary motor cortex, premotor cortex, and supplementary motor area) and parietal lobe (posterior parietal cortex and primary somatosensory cortex) (Campbell, 1905). The CST, which was associated with a higher Gold-MSI score in our study, is one of the main pyramidal tracts and projects from the motor cortex to lower motor neurons in the spinal cord, which regulates the movements of limbs and trunk (Figure 5). Cortico-pontine tracts are bundles that arise from each lobe cortex [e.g., fronto-pontine (associated with higher active engagement in our study), parieto-pontine, and etc.] and terminate in pontine nuclei (Figure 5; Rea, 2015). The cortico-pontine tracts allow the coordination of motor functions by communicating with the opposite cerebellum which was associated with the higher active engagement scores of the Gold-MSI in our study. This communication takes places through the MCP which appeared to be associated with higher Gold-MSI scores in our study (Rea, 2015). This result is consistent with previous studies depicting higher cerebellar volume in musicians relative to non-musicias (Hutchinson et al., 2003). The same study also reported a positive correlation between relative cerebellar volume and lifelong intensity of musical practice which represents structural adaptation to long-term motor and cognitive functional demands in the cerebellum. A circuitry model that explains the motor system interconnections mentions that the cortico-basal ganglia-thalamo-cortical loop is a neural circuit system with both inhibitory and excitatory fibers (Figure 5; Silkis, 2001): Cortical inputs into the basal ganglia and thalamic inputs into the cortex are excitatory, whereas the basal ganglia outputs to the thalamus are inhibitory. Additionally, the basal ganglia and cerebellum, which their microstructural alterations were associated with higher scores of active engagement subscale in our study, modulate the output of the CST. CST which was also shown to be associated with higher Gold-MSI score. Along with other descending motor neurons and they receive inputs from the motor and somatosensory cortex, brain stem, and spinal cord, and consecutively project back to the motor cortex through thalamus (Figure 5).

Our findings show a significant association between the CC with higher Gold-MSI scores in our male participants, and conversely, lower scores in female participants. The CC is the main commissural bundle in brain which interconnects the two contralateral lobes. The AF that was shown to be associated with higher Gold-MSI score in our study, is traditionally known for its critical role in language processing functions music development, interconnects the two main language processing areas Broca’s area in the inferior frontal gyrus and Wernicke’s area in the posterior-superior temporal gyrus (Eichert et al., 2019). The AF is confirmed to be responsible for some clinical disorders, including conduction aphasia (Acharya and Maani, 2020), tone-deafness (Loui et al., 2009), and stuttering (Cieslak et al., 2015). The cingulum, that was shown to be associated with a higher Gold-MSI score in our study, is the main component of the limbic system. The cingulum is a fiber bundle beneath the cingulate cortex interconnecting the frontal lobe with the temporal and parietal lobes closely above the CC (Bruni and Montemurro, 2009). Ultimately, it seems that fiber bundles with close functional connection with the motor system might play a significant role in both active engagement and musical training subscales of the Gold-MSI. Previous studies have also followed our findings and reported the increased GM volume in the primary motor cortex, premotor cortex, somatosensory areas, parietal cortex, prefrontal cortex, and cerebellum in musicians (Gaser and Schlaug, 2003; Han et al., 2009; Lai et al., 2012; Acer et al., 2018; Oechslin et al., 2018). Apart from similar studies that investigated GM alterations, previous efforts have been taken to address the WM differences mainly between musicians and non-musicians using DTI. For instance, through the investigation of DTI human studies on musical perception, the AF was recognized as a bundle of WM that is responsible for language and music functions (Loui and Schlaug, 2009), and a lower WM integrity in AF was observed in musically tone-deaf individuals (Loui et al., 2009). Besides, autistic children were reported to have a lower FA value in left AF compared to controls (Lai et al., 2012). In a study done in a Mandarin population with amusia, DTI-derived results showed the higher WM integrity in the right posterior AF as well as lower WM integrity in the right anterior AF in amusics (Chen et al., 2018). Moreover, increased FA value in the right AF was observed as the effect of musical training on the WM changes in a short time (20 min, three times per week, for 4 weeks), compared to controls (Moore et al., 2017). Above all, the higher integrity and volume of right AF are reported to have an association with music learning speed and rate (Vaquero et al., 2018). Therefore, on top of the aforementioned findings, our study also suggests the higher WM coherence in AF to have a significant connection with musical sophistication.

In line with our findings, CC changes have been shown to be different among individuals with different musical abilities. Importantly, our results found a marginally significant correlation between musical training and QA values of CC in males. Even so, our findings notably demonstrated that WM integrity changes in CC were significantly different between genders, being positive in males and negative in females. This difference could potentially arise from the impact of “age of onset” of musical training, which may have been different across our groups of participants. Nonetheless, finding from several DTI studies support our results related to CC changes associated with musical training. For instance, seminal study which investigated the differences in musicians and non-musicians’ brain structure, found the anterior part of the CC to be larger in musicians (Schlaug et al., 1995). Another study also deduced that intensive and professional musical training since childhood leads to significant changes in WM architecture (Schmithorst and Wilke, 2002). A more recent study examined the association between musical perceptional abilities, assessed by the Profile of Music Perception Skills (PROMS) and WM microstructure using DTI, and reported specific parts of the CC to be significantly involved with musicianship (Rajan et al., 2019). WM plasticity in early trained musicians was also detected as the higher WM integrity in posterior mid-body and isthmus of CC in them (Steele et al., 2013). Another longitudinal study compared the children with more than 2 years of musical training with non-trained controls and reported the highest WM integrity (FA value) in the CC in trained children, particularly in the crossing bundles interconnecting superior frontal, sensory, and motor areas (Habibi et al., 2018). Moreover, professional drummers showed to have higher microstructural diffusion properties in the CC than non-musical controls (Schlaffke et al., 2020). These microstructural changes in the CC among the musicians are probably owing to the bimanual coordination –the ability to simultaneously control multiple movements– that playing a musical instrument demands (Swinnen, 2002; Palomar-García et al., 2017).

Our results also show a positive association between the CST microstructural alterations with higher scores in both GOLD-MSI scores in males, but not in females. The maturation of CST fibers has been shown to correspond to the improvement of fine finger movements (Paus et al., 1999). The differences of diffusion parameters in the CST of professional musicians and non-musicians investigations suggested a lower WM integrity in the CST and plastic changes in WM in professional musicians compared to non-musicians (Imfeld et al., 2009). Besides, a higher WM integrity in the CST, superior longitudinal fasciculus and the CC was reported in dancers comparing to musicians in a previous DTI survey which investigated the WM alteration between dancers and musicians based on their different required motor functions (Giacosa et al., 2016). Moreover, the tract volume and number of streamlines of superior and middle cerebellar peduncles, which were associated with higher Gold-MSI score in our study, were previously reported to be higher in musicians compared to non-musicians (Abdul-Kareem et al., 2011). Another recent study evaluated the GM and WM alterations between professional musicians and non-musicians and found the lower WM coherence in CC, superior longitudinal fasciculus, forceps major and minor, and right AF but higher FA value in right CST as well as increased GM volume in bilateral cerebellar hemispheres, supramarginal and angular gyrus, left parietal lobule, and left temporal lobe in the professional musicians compared to non-musicians control group (Acer et al., 2018). Thus, given the significant role of CST in the motor system, and sufficient pieces of evidence suggesting the significant CST WM differences in musicians, we can conclude that our results showing WM changes in CST and its correlation with both musical sophistication subscales is consistent with formerly existing evidence.

Although this study followed an exploratory approach for investigating WM microstructural alterations links with musicality, we hypothesized that there could be potential sex differences in terms of these alterations and associations with active musical engagement and training. In light of the ample evidence of the gender differences in brain structures using traditional or voxel-based morphometry (Amunts et al., 2000; Nopoulos et al., 2000; Good et al., 2001), and with specific attention to various cognitive domains (Hyde, 2016), we aimed to investigate the potential gender differences in musical sophistication. In line with our hypothesis, we found WM microstructural differences in males compared to females in both investigated GOLD-MSI subscales. Notably, limited studies have addressed the gender differences in the field of music. For instance, auditory processing was reported to be different in males comparing to females in animal studies (Yoder et al., 2015). Besides, the anterior CC was observed to be significantly larger in male musicians in comparison to non-musicians; however, this structural change was not observed in females equivalent groups (Lee et al., 2003). Furthermore, relatively similar to our findings in both genders, music processing was reported to be conducted bilaterally, with right dominance in females’ and males’ hemispheres (Koelsch et al., 2003). These pieces of evidence derived from neural data are also consistent with findings from a study that shows female advantages at recognizing familiar melodies stemming from their superiority in declarative memory and behavioral sex differences in higher-level aspects of musical cognition (Miles et al., 2016).

Our findings should be interpreted in light of some limitations. These findings only specify a number of WM tracts with significant association with the variable of interest, yet the causal association remains unclear. Thus, it remains unanswered whether the musical engagement or training leads to differences in WM tracts or vice versa; and if some genetic or non-genetic etiologic factors result in these WM microstructural alterations and make them predisposing factors for musical sophistication. With respect to our findings about sex differences in WM alterations related to musical sophistication, our study could have benefitted from more measurements of musical training for detecting potential confounding factors such as age of onset of musical training or musical training intensity. Additionally, it is noteworthy to mention that dMRI connectometry is a novel WM structural analytic technique to address the WM connectivity of specific bundle fibers, but the functional features of those trajectories are failed to address. Hence, further multimodal imaging modalities are expected to rectify this crucial knowledge gap (Burunat et al., 2015).

This study not only provides a basis for the investigation of differences regarding musical skills and talent, but also serves as a potential for further studies in the etiology, prevention, and management of clinical conditions associated with problems in comprehension and processing of information in the aforementioned brain structures. Future studies could take into account the factors such as age of onset of musical training, musical training intensity, and different aspects of auditory processing and their association with WM microstructural alterations. In addition, our sample was precisely controlled for potential neurological, psychological, and neurocognitive confounding factors; however, future studies may explore ethnical and/or cultural aspects of musical sophistication and their associated neural alterations, which may exhibit contradictory results relative to our findings that took place on a population from a WEIRD society (Hoffman et al., 2011). Finally, it is worth mentioning that we employed two specific subscales of the GOLD-MSI, including active musical engagement and musical training, to explore their correlations with WM microstructural alterations and potential sex differences in this regard; but the above mentioned association with three other subscales of GOLD-MSI (i.e., self-reported perceptual abilities, self-reported singing abilities, and sophisticated emotional engagement with music) could be further investigated to unravel which WM tracts and their connectivity are associated with those aspects of musical sophistication and potential sex differences.

## Conclusion

In conclusion, our study is the first dMRI study investigation that explored the brain microstructural alterations related to musical sophistication in a healthy population. Our findings pointed to the notion that WM microstructures with functional connection with motor and somatosensory areas (such as the corpus callosum, corticospinal tract, cingulum, cerebellar peduncle, parieto-pontine tract, and cerebellum), and language processing areas (such as the arcuate fasciculus) have a significant correlation with active musical engagement and training. Although some caution is warranted since this is the first study that investigated sex differences in brain’s white matter microstructural alterations related to musical sophistication using a novel approach, significant sex differences were observed indicating the corpus callosum to be the only WM tract associated with musical sophistication in females, while a wide range of WM microstructures were shown to be linked with this ability in males. Our results are consistent with the idea that the coordination between auditory and motor systems is necessary for music performance, particularly musical active engagement and training.

## Data Availability Statement

The original contributions presented in the study are included in the article/supplementary material, further inquiries can be directed to the corresponding author/s.

## Ethics Statement

The present study was carried out in accordance with the World Medical Association Declaration of Helsinki revised in 1989 and approved by the Ethics Committee of the University of Leipzig (reference number 154/13-ff).

## Author Contributions

MA and PR conceived of the presented idea. MA performed the computations and data analysis. M-MM, PR, HS, and ZS wrote the first draft of the manuscript. All authors discussed the results and contributed to the final manuscript.

## Conflict of Interest

The authors declare that the research was conducted in the absence of any commercial or financial relationships that could be construed as a potential conflict of interest.

## Footnotes

1 http://doi.org/10.15387/fcp_indi.mpi_lemon

2 http://www.exploredti.com

## References

[B1] Abdul-Kareem I. A. Stancak A. Parkes L. M. Sluming V. (2011). Increased gray matter volume of left pars opercularis in male orchestral musicians correlate positively with years of musical performance. *J. Magn. Resonance Imag.* 33 24–32. 10.1002/jmri.22391 21182117

[B2] Acer N. Bastepe-Gray S. Sagiroglu A. Gumus K. Z. Degirmencioglu L. Zararsiz G. (2018). Diffusion tensor and volumetric magnetic resonance imaging findings in the brains of professional musicians. *J. Chem. Neuroanatomy* 88 33–40. 10.1016/j.jchemneu.2017.11.003 29113947

[B3] Acharya A. B. Maani C. V. (2020). *Conduction Aphasia. in StatPearls [Internet].* Treasure Island, FL: StatPearls.

[B4] Amunts K. Jäncke L. Mohlberg H. Steinmetz H. Zilles K. (2000). Interhemispheric asymmetry of the human motor cortex related to handedness and gender. *Neuropsychologia* 38 304–312. 10.1016/s0028-3932(99)00075-5 10678696

[B5] Babayan A. Erbey M. Kumral D. Reinelt J. D. Reiter A. M. Röbbig J. (2019). A mind-brain-body dataset of MRI, EEG, cognition, emotion, and peripheral physiology in young and old adults. *Sci. Data* 6 1–21. 30747911 10.1038/sdata.2018.308 PMC6371893

[B6] Baker D. J. Ventura J. Calamia M. Shanahan D. Elliott E. M. (2020). Examining musical sophistication: a replication and theoretical commentary on the Goldsmiths Musical Sophistication Index. *Musicae Sci.* 24 411–429. 10.1177/1029864918811879

[B7] Boyle J. D. Radocy R. E. (1987). *Measurement and Evaluation of Musical Experiences.* New Yoerk, NY: Schirmer Books.

[B8] Brockmeier S. Fitzgerald D. Searle O. Fitzgerald H. Grasmeder M. Hilbig S. (2011). The MuSIC perception test: a novel battery for testing music perception of cochlear implant users. *Cochlear Implants Int.* 12 10–20. 10.1179/146701010x12677899497236 21756454

[B9] Bruni J. E. Montemurro D. G. (2009). *Human Neuroanatomy: A Text, Brain Atlas, and Laboratory Dissection Guide.* Oxford: Oxford University Press.

[B10] Burkhard A. Hänggi J. Elmer S. Jäncke L. (2020). The importance of the fibre tracts connecting the planum temporale in absolute pitch possessors. *NeuroImage* 211:116590. 10.1016/j.neuroimage.2020.116590 32004719

[B11] Burunat I. Brattico E. Puoliväli T. Ristaniemi T. Sams M. Toiviainen P. (2015). Action in perception: prominent visuo-motor functional symmetry in musicians during music listening. *PLoS One* 10:e0138238. 10.1371/journal.pone.0138238 26422790 PMC4589413

[B12] Campbell A. W. (1905). *Histological Studies on the Localisation of Cerebral Function.* Cambridge: Cambridge University Press.

[B13] Chen X. Zhao Y. Zhong S. Cui Z. Li J. Gong G. (2018). The lateralized arcuate fasciculus in developmental pitch disorders among mandarin amusics: left for speech and right for music. *Brain Struct. Funct.* 223 2013–2024. 29322239 10.1007/s00429-018-1608-2

[B14] Cieslak M. Ingham R. J. Ingham J. C. Grafton S. T. (2015). Anomalous white matter morphology in adults who stutter. *J. Speech Lang. Hearing Res.* 58 268–277. 10.1044/2015_jslhr-s-14-0193 PMC4675119 25635376

[B15] Eichert N. Verhagen L. Folloni D. Jbabdi S. Khrapitchev A. A. Sibson N. R. (2019). What is special about the human arcuate fasciculus? Lateralization, projections, and expansion. *Cortex* 118 107–115. 10.1016/j.cortex.2018.05.005 29937266 PMC6699597

[B16] Elmer S. Hänggi J. Meyer M. Jäncke L. (2013). Increased cortical surface area of the left planum temporale in musicians facilitates the categorization of phonetic and temporal speech sounds. *Cortex* 49 2812–2821. 10.1016/j.cortex.2013.03.007 23628644

[B17] Gaser C. Schlaug G. (2003). Brain structures differ between musicians and non-musicians. *J. Neurosci.* 23 9240–9245. 10.1523/jneurosci.23-27-09240.2003 14534258 PMC6740845

[B18] Giacosa C. Karpati F. J. Foster N. E. Penhune V. B. Hyde K. L. (2016). Dance and music training have different effects on white matter diffusivity in sensorimotor pathways. *Neuroimage* 135 273–286. 10.1016/j.neuroimage.2016.04.048 27114054

[B19] Good C. D. Johnsrude I. Ashburner J. Henson R. N. Friston K. J. Frackowiak R. S. (2001). Cerebral asymmetry and the effects of sex and handedness on brain structure: a voxel-based morphometric analysis of 465 normal adult human brains. *Neuroimage* 14 685–700. 10.1006/nimg.2001.0857 11506541

[B20] Greenberg D. M. Müllensiefen D. Lamb M. E. Rentfrow P. J. (2015). Personality predicts musical sophistication. *J. Res. Pers.* 58 154–158. 10.1016/j.jrp.2015.06.002

[B21] Habibi A. Damasio A. Ilari B. Veiga R. Joshi A. A. Leahy R. M. (2018). Childhood music training induces change in micro and macroscopic brain structure: results from a longitudinal study. *Cereb. Cortex* 28 4336–4347. 10.1093/cercor/bhx286 29126181

[B22] Halwani G. F. Loui P. Rüber T. Schlaug G. (2011). Effects of practice and experience on the arcuate fasciculus: comparing singers, instrumentalists, and non-musicians. *Front. Psychol.* 2:156. 10.3389/fpsyg.2011.00156 21779271 PMC3133864

[B23] Han Y. Yang H. Lv Y.-T. Zhu C.-Z. He Y. Tang H.-H. (2009). Gray matter density and white matter integrity in pianists’ brain: a combined structural and diffusion tensor MRI study. *Neurosci. Lett.* 459 3–6. 10.1016/j.neulet.2008.07.056 18672026

[B24] Hoffman M. Gneezy U. List J. A. (2011). Nurture affects gender differences in spatial abilities. *Proc. Natl. Acad. Sci. U.S.A.* 108:14786. 10.1073/pnas.1015182108 21876159 PMC3169128

[B25] Hutchinson S. Lee L. H. Gaab N. Schlaug G. (2003). Cerebellar volume of musicians. *Cereb. Cortex* 13 943–949. 10.1093/cercor/13.9.943 12902393

[B26] Hyde J. S. (2016). Sex and cognition: gender and cognitive functions. *Curr. Opin. Neurobiol.* 38 53–56. 10.1016/j.conb.2016.02.007 26953847

[B27] Imfeld A. Oechslin M. S. Meyer M. Loenneker T. Jancke L. (2009). White matter plasticity in the corticospinal tract of musicians: a diffusion tensor imaging study. *Neuroimage* 46 600–607. 10.1016/j.neuroimage.2009.02.025 19264144

[B28] Ingalhalikar M. Smith A. Parker D. Satterthwaite T. D. Elliott M. A. Ruparel K. (2014). Sex differences in the structural connectome of the human brain. *Proc. Natl. Acad. Sci. U.S.A.* 111:823. 24297904 10.1073/pnas.1316909110 PMC3896179

[B29] Keenan J. P. Thangaraj V. Halpern A. R. Schlaug G. (2001). Absolute pitch and planum temporale. *Neuroimage* 14 1402–1408. 10.1006/nimg.2001.0925 11707095

[B30] Koelsch S. Maess B. Grossmann T. Friederici A. D. (2003). Electric brain responses reveal gender differences in music processing. *Neuroreport* 14 709–713. 10.1097/00001756-200304150-00010 12692468

[B31] Lai G. Pantazatos S. P. Schneider H. Hirsch J. (2012). Neural systems for speech and song in autism. *Brain* 135 961–975. 10.1093/brain/awr335 22298195 PMC3286324

[B32] Larrouy-Maestri P. Harrison P. M. Müllensiefen D. (2019). The mistuning perception test: a new measurement instrument. *Behav. Res. Methods* 51 663–675. 10.3758/s13428-019-01225-1 30924106 PMC6478636

[B33] Law L. N. Zentner M. (2012). Assessing musical abilities objectively: construction and validation of the Profile of Music Perception Skills. *PLoS One* 7:e52508. 10.1371/journal.pone.0052508 23285071 PMC3532219

[B34] Lee D. J. Chen Y. Schlaug G. (2003). Corpus callosum: musician and gender effects. *Neuroreport* 14 205–209. 10.1097/00001756-200302100-00009 12598730

[B35] Leemans A. Jeurissen B. Sijbers J. Jones D. (2009). “ExploreDTI: a graphical toolbox for processing, analyzing, and visualizing diffusion MR data,” in *Proceedings of the International Society for Magnetic Resonance in Medicine*, (Honolulu).

[B36] Levitin D. J. (2012). What does it mean to be musical? *Neuron* 73 633–637. 10.1016/j.neuron.2012.01.017 22365540

[B37] Li X. Zatorre R. J. Du Y. (2021). The microstructural plasticity of the arcuate fasciculus undergirds improved speech in noise perception in musicians. *Cereb. Cortex* bhab063. [Online ahead of print]. 10.1093/cercor/bhab063 34037726 PMC8328222

[B38] Loui P. Alsop D. Schlaug G. (2009). Tone deafness: a new disconnection syndrome? *J. Neurosci.* 29 10215–10220. 10.1523/jneurosci.1701-09.2009 19692596 PMC2747525

[B39] Loui P. Schlaug G. (2009). Investigating musical disorders with diffusion tensor imaging: a comparison of imaging parameters. *Ann. N. Y. Acad. Sci.* 1169:121. 10.1111/j.1749-6632.2009.04781.x 19673766 PMC2785849

[B40] Mehrabinejad M.-M. Sanjari Moghaddam H. Mohammadi E. Hajighadery A. Sinaeifar Z. Aarabi M. H. (2021). Sex differences in microstructural white matter alterations of mathematics anxiety based on diffusion MRI connectometry. *Neuropsychology* 35:197. 10.1037/neu0000684 33764110

[B41] Merriam A. P. Merriam V. (1964). *The Anthropology of Music.* Evanston, IL: Northwestern University Press.

[B42] Miles S. A. Miranda R. A. Ullman M. T. (2016). Sex differences in music: a female advantage at recognizing familiar melodies. *Front. Psychol.* 7:278. 10.3389/fpsyg.2016.00278 26973574 PMC4771742

[B43] Moghaddam H. S. Mehrabinejad M.-M. Mohebi F. Hajighadery A. Maroufi S. F. Rahimi R. (2020). Microstructural white matter alterations and personality traits: a diffusion MRI study. *J. Res. Personal.* 88:104010. 10.1016/j.jrp.2020.104010

[B44] Møller C. Garza-Villarreal E. A. Hansen N. C. Højlund A. Bærentsen K. B. Chakravarty M. M. (2021). Audiovisual structural connectivity in musicians and non-musicians: a cortical thickness and diffusion tensor imaging study. *Sci. Rep.* 11 1–14. 33619288 10.1038/s41598-021-83135-x PMC7900203

[B45] Moore E. Schaefer R. S. Bastin M. E. Roberts N. Overy K. (2017). Diffusion tensor MRI tractography reveals increased fractional anisotropy (FA) in arcuate fasciculus following music-cued motor training. *Brain Cogn.* 116 40–46. 10.1016/j.bandc.2017.05.001 28618361 PMC5479403

[B46] Müllensiefen D. Gingras B. Musil J. Stewart L. (2014). The musicality of non-musicians: an index for assessing musical sophistication in the general population. *PLoS One* 9:e89642. 10.1371/journal.pone.0089642 24586929 PMC3935919

[B47] Nopoulos P. Flaum M. O’leary D. Andreasen N. C. (2000). Sexual dimorphism in the human brain: evaluation of tissue volume, tissue composition and surface anatomy using magnetic resonance imaging. *Psychiatry Res. Neuroimag.* 98 1–13. 10.1016/s0925-4927(99)00044-x 10708922

[B48] Oechslin M. S. Gschwind M. James C. E. (2018). Tracking training-related plasticity by combining fMRI and DTI: the right hemisphere ventral stream mediates musical syntax processing. *Cereb. Cortex* 28 1209–1218. 10.1093/cercor/bhx033 28203797

[B49] Palomar-García M. Zatorre R. J. Ventura-Campos N. Bueichekú E. Ávila C. (2017). Modulation of functional connectivity in auditory-motor networks in musicians compared with nonmusicians. *Cereb. Cortex* 27 2768–2778. 27166170 10.1093/cercor/bhw120

[B50] Parsons L. M. (2001). Exploring the functional neuroanatomy of music performance, perception, and comprehension. *Ann. N. Y. Acad. Sci.* 930 211–231. 10.1111/j.1749-6632.2001.tb05735.x 11458831

[B51] Paus T. Zijdenbos A. Worsley K. Collins D. L. Blumenthal J. Giedd J. N. (1999). Structural maturation of neural pathways in children and adolescents: *in vivo* study. *Science* 283 1908–1911. 10.1126/science.283.5409.1908 10082463

[B52] Rahmani F. Sobhani S. Aarabi M. H. (2017). Sequential language learning and language immersion in bilingualism: diffusion MRI connectometry reveals microstructural evidence. *Exp. Brain Res.* 235 2935–2945. 10.1007/s00221-017-5029-x 28702836

[B53] Rajan A. Valla J. M. Alappatt J. A. Sharda M. Shah A. Ingalhalikar M. (2019). Wired for musical rhythm? A diffusion MRI-based study of individual differences in music perception. *Brain Struct. Funct.* 224 1711–1722. 10.1007/s00429-019-01868-y 30949766

[B54] Rea P. (2015). *Essential Clinical Anatomy of the Nervous System.* Cambridge, MA: Academic Press.

[B55] Rickard N. S. Chin T. Coutinho E. Scherer K. R. (2015). “The MUSEBAQ: a comprehensive and modular instrument for assessing musical engagement,” in *Proceedings of the 4th International Conference on Music and Emotion (ICME’4)*, (Geneva).

[B56] Rüber T. Lindenberg R. Schlaug G. (2015). Differential adaptation of descending motor tracts in musicians. *Cereb. Cortex* 25 1490–1498. 10.1093/cercor/bht331 24363265 PMC4428294

[B57] Sato K. Kirino E. Tanaka S. (2015). A voxel-based morphometry study of the brain of university students majoring in music and nonmusic disciplines. *Behav. Neurol.* 2015:274919. 26494943 10.1155/2015/274919 PMC4606127

[B58] Schaal N. K. Bauer A.-K. R. Müllensiefen D. (2014). Der Gold-MSI: replikation und validierung eines fragebogeninstrumentes zur messung musikalischer erfahrenheit anhand einer deutschen stichprobe. *Musicae Sci.* 18 423–447. 10.1177/1029864914541851

[B59] Schlaffke L. Friedrich S. Tegenthoff M. Güntürkün O. Genç E. Ocklenburg S. (2020). Boom Chack Boom—A multimethod investigation of motor inhibition in professional drummers. *Brain Behav.* 10:e01490. 31801182 10.1002/brb3.1490 PMC6955843

[B60] Schlaug G. (2015). Musicians and music making as a model for the study of brain plasticity. *Prog. Brain Res.* 217 37–55. 10.1016/bs.pbr.2014.11.020 25725909 PMC4430083

[B61] Schlaug G. Jäncke L. Huang Y. Staiger J. F. Steinmetz H. (1995). Increased corpus callosum size in musicians. *Neuropsychologia* 33 1047–1055. 10.1016/0028-3932(95)00045-5 8524453

[B62] Schmithorst V. J. Wilke M. (2002). Differences in white matter architecture between musicians and non-musicians: a diffusion tensor imaging study. *Neurosci. Lett.* 321 57–60. 10.1016/s0304-3940(02)00054-x 11872256

[B63] Schneider P. Scherg M. Dosch H. G. Specht H. J. Gutschalk A. Rupp A. (2002). Morphology of Heschl’s gyrus reflects enhanced activation in the auditory cortex of musicians. *Nat. Neurosci.* 5 688–694. 10.1038/nn871 12068300

[B64] Seashore C. E. Lewis D. Saetveit J. G. (1956). *Seashore Measures of Musical Talents.* Oxford: Psychological Corp.

[B65] Silkis I. (2001). The cortico-basal ganglia-thalamocortical circuit with synaptic plasticity. II. Mechanism of synergistic modulation of thalamic activity via the direct and indirect pathways through the basal ganglia. *Biosystems* 59 7–14. 10.1016/s0303-2647(00)00135-0 11226622

[B66] Sluming V. Barrick T. Howard M. Cezayirli E. Mayes A. Roberts N. (2002). Voxel-based morphometry reveals increased gray matter density in Broca’s area in male symphony orchestra musicians. *Neuroimage* 17 1613–1622. 10.1006/nimg.2002.1288 12414299

[B67] Steele C. J. Bailey J. A. Zatorre R. J. Penhune V. B. (2013). Early musical training and white-matter plasticity in the corpus callosum: evidence for a sensitive period. *J. Neurosci.* 33 1282–1290. 10.1523/jneurosci.3578-12.2013 23325263 PMC6704889

[B68] Swinnen S. P. (2002). Intermanual coordination: from behavioural principles to neural-network interactions. *Nat. Rev. Neurosci.* 3 348–359. 10.1038/nrn807 11988774

[B69] van Vugt F. T. Hartmann K. Altenmüller E. Mohammadi B. Margulies D. S. (2021). The impact of early musical training on striatal functional connectivity. *NeuroImage* 238:118251. 10.1016/j.neuroimage.2021.118251 34116147

[B70] Vaquero L. Ramos-Escobar N. François C. Penhune V. Rodríguez-Fornells A. (2018). White-matter structural connectivity predicts short-term melody and rhythm learning in non-musicians. *NeuroImage* 181 252–262. 10.1016/j.neuroimage.2018.06.054 29929006

[B71] Wan C. Y. Zheng X. Marchina S. Norton A. Schlaug G. (2014). Intensive therapy induces contralateral white matter changes in chronic stroke patients with Broca’s aphasia. *Brain Lang.* 136 1–7. 10.1016/j.bandl.2014.03.011 25041868 PMC4425280

[B72] Wu J. Zhang J. Ding X. Li R. Zhou C. (2013). The effects of music on brain functional networks: a network analysis. *Neuroscience* 250 49–59. 10.1016/j.neuroscience.2013.06.021 23806719

[B73] Yeh F.-C. Badre D. Verstynen T. (2016). Connectometry: a statistical approach harnessing the analytical potential of the local connectome. *Neuroimage* 125 162–171. 10.1016/j.neuroimage.2015.10.053 26499808

[B74] Yeh F.-C. Verstynen T. D. Wang Y. Fernández-Miranda J. C. Tseng W.-Y. I. (2013). Deterministic diffusion fiber tracking improved by quantitative anisotropy. *PLoS One* 8:e80713. 10.1371/journal.pone.0080713 24348913 PMC3858183

[B75] Yeh F.-C. Wedeen V. J. Tseng W.-Y. I. (2011). Estimation of fiber orientation and spin density distribution by diffusion deconvolution. *Neuroimage* 55 1054–1062. 10.1016/j.neuroimage.2010.11.087 21232611

[B76] Yoder K. M. Phan M. L. Lu K. Vicario D. S. (2015). He hears, she hears: are there sex differences in auditory processing? *Dev. Neurobiol.* 75 302–314. 10.1002/dneu.22231 25220950 PMC4324369

